# Mapping the branching pattern of the middle cerebral artery in the camel (*Camelus dromedarius*): a comprehensive anatomical analysis

**DOI:** 10.3389/fvets.2023.1224197

**Published:** 2023-07-11

**Authors:** Ahmad Al Aiyan, Rinsha Balan

**Affiliations:** Department of Veterinary Medicine, College of Agriculture and Veterinary Medicine, United Arab Emirates University, Al Ain, United Arab Emirates

**Keywords:** dromedary camel, brain, middle cerebral artery, circle of Willis, cerebral arteries

## Abstract

The complex branching structure of the middle cerebral artery serves as a crucial component in the blood supply to the cerebral cortex, playing a key role in sustaining brain function and overall neurological health in mammals. A thorough understanding of the branching structure of the middle cerebral artery is required for the advancement of veterinary medicine and neuroscience research. In this study, we provide the first comprehensive anatomical analysis of the branching structure of the middle cerebral artery (MCA) in the dromedary brain. To date, no study has examined the MCA branches in dromedaries. By examining 80 cerebral hemispheres from freshly slaughtered male dromedary camels aged 2–6  years, we aimed to explain the origin, course, and branching patterns of the MCA in the dromedary camel. Advanced casting techniques using colored latex, epoxy paint, and liquid plastic have been used to create precise renderings of the MCA structure. Our findings revealed that the MCA is the principal branch of the rostral cerebral artery and serves as the primary blood supply to the telencephalon in dromedaries. The main trunk of the MCA splits into several cortical branches, each supplying blood to a specific cerebral hemisphere. These branches comprise the rostral and caudal olfactory arteries; orbital artery; superior, middle, and inferior frontal arteries; rostral, middle, and caudal parietal arteries; and dorsal, middle, and ventral temporal arteries. This groundbreaking work considerably advances our understanding of the dromedary cerebrovascular system by providing insightful information on the anatomy and topography of the MCA. Our findings open new avenues for advancements in veterinary medicine and neuroscience research, with potential applications in the diagnosis and treatment of neurological disorders in dromedary camels. Furthermore, understanding the unique branching pattern of the MCA may have implications for comparative neuroanatomy and the evolution of cerebrovascular systems across species.

## 1. Introduction

The one-humped camel, also referred to as the dromedary, belongs to the Camelidae family and is native to the desert areas of the Middle East and North Africa. Due to its long history of physiological adaptation to harsh desert environment, this species is a particularly interesting subject to study in terms of both its evolutionary history and its physiological adaptations.

**Figure 1:**
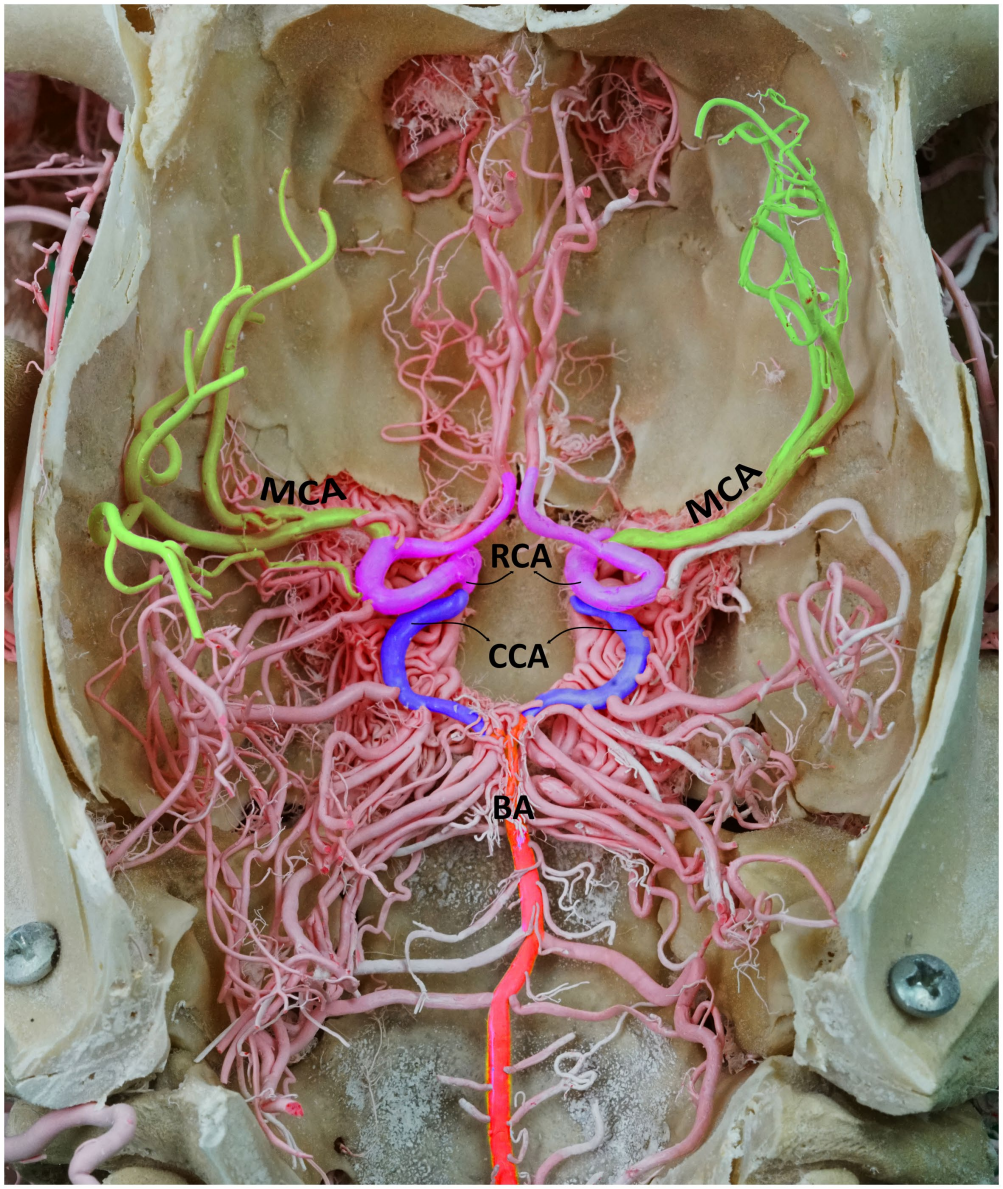
Dorsal perspective of a macerated camel skull, showcasing the arterial supply to the dromedary brain. BA, basilar artery; CCA, caudal communicating artery; RCA, rostral cerebral artery; MCA, middle cerebral artery. Note that the RERM is located ventral to the cerebral arterial circle within the cavernous sinus at the base of the cranial cavity.

Because of the excessive metabolic needs of nerve cells, the central nervous system is acutely dependent on the constant flow of oxygenated blood. Different afferent systems combine blood output before supplying blood to the brain. This process occurs via the circle of Willis, also known as the cerebral arterial circle (CAC) (Figure 1). The CAC is a complex network of arteries at the base of the skull that connects the carotid and vertebrobasilar systems and allows blood from these two systems to combine (1–3).

A pair of major arteries, known as the rostral cerebral artery (RCA) and caudal communicating artery (CCA), emerges from the rostral epidural rete mirabile (RERM), forming the circle of Willis after joining the basilar artery. Several branches leave the CAC and innervate various regions of the dromedary brain (4, 5). The middle cerebral artery (MCA) has extensive clinical significance and is the most commonly pathologically affected blood vessel in the brain. Stroke, aneurysm formation, and embolism are some of the most prevalent pathologies (6, 7).

Despite being a significant blood channel that supplies blood to the brain, to our knowledge, there is no study on the cortical branches of the MCA in dromedaries. The cortical branches of the MCA have been the subject of only a few studies in selected animal species, including domestic pigs (8), bison (9), roe deer (10), elks (11), goats (12), and cattle (13).

Several authors have referred to multiple MCA as arterial varieties, including those found in camelids (14), horses (15, 16), roe deer (17), and cattle (18). Despite reaching the same regions of the cerebrum in the aforementioned animal species, the cortical branches of the MCA differ in their descent and division patterns. Sulcus coverage of the cortex has an impact on how the MCA is formed, since a particular configuration of the sulci on the mammalian cortical surface can affect the MCA’s division pattern. For instance, deeper or more complex sulcal patterns may necessitate more intricate branching of the MCA to adequately supply the brain tissue (19).

Preliminary findings of our ongoing research on the gyri and sulci of the camel brain, which is not yet published, suggest a strong correlation between sulcal configuration and MCA branching patterns (Al Aiyan et al., unpublished)^1^.

The present study aimed to provide a comprehensive description of the arterial branches of the MCA in dromedaries. By employing improved epoxy paint casting techniques, we generated precise three-dimensional representations and highlighted minor branches that would have been missed during dissection using other techniques. Understanding the unique branching pattern of the MCA may have implications for comparative neuroanatomy and the evolution of cerebrovascular systems across species and is essential for advancements in neuroscience research, with potential applications in the diagnosis and treatment of neurological disorders in dromedaries.

## 2. Materials and methods

This study followed the research ethics policy and was approved by the Animal Research Ethics Committee of the United Arab Emirates University (ERA 2019 5,850). In this study, 40 brains (a total of 80 cerebral hemispheres) of freshly slaughtered male Omani dromedaries aged 2–6 years were obtained from Al Ain City Municipality Camel Slaughterhouses. The cerebral arteries were cast by injecting various casting materials through the right and left common carotid arteries.

Liquid polyurethane resin (Polytek EasyFlo 60 Liquid Plastic) was injected into ten heads, and red epoxy resin (Gulfguard Epoxy—Color 04E53) was injected into another ten heads. Then, all 20 camel heads were injected with red latex neoprene solution (Latex Globalsil AL 20, Global Chemica S.R.I). After casting, the heads were maintained at 5°C for 1–2 days until the arteries had palpable hardness. The heads were then dissected to remove the surrounding skin and muscles. The roof of the cranium and vertebrae were sliced open with a rotating power saw. The brain of the latex-injected samples was carefully extracted from the cranial cavity. The cranial cavity of the liquid polyurethane resin-injected samples was washed using a high-pressure washer. This process washed away the brain tissues without affecting the resin cast fillers. The red epoxy resin-injected samples were subjected to a slower process of chemical digestion by immersing the craniotomized heads in 5% potassium hydroxide, which digested all soft tissues and bones but not the epoxy resin in the arteries. This process took ab to 3 weeks to get the cast. Additional information regarding the casting methods employed can be found in our earlier studies (4, 5).

The selection of different casting materials for the injections was a strategic decision based on the specific challenges of our experimental study. Liquid polyurethane resin (Polytek EasyFlo 60 Liquid Plastic) was chosen due to its ability to produce a solid but slightly flexible cast. This flexibility allows little movements of the branches, lowering the risk of breakage and assuring the preservation of the complicated artery network. On the other hand, epoxy resin (Gulfguard Epoxy—Color 04E53) was utilized to create a highly rigid cast. This property was ideal for gaining a detailed 3D representation of the studied arteries. However, this kind of resin lack of flexibility proposed that the arteries and branches were more subject to breaking. In addition, in order to remove the brains while protecting the arteries, we also utilized a red latex neoprene solution (Latex Globalsil AL 20, Global Chemica S.R.I). This material offers high flexibility, which was advantageous for this particular step of the process.

All samples were photographed using a Sony a7R II at a setting of 42 megapixels for Fine JPEG. Using Adobe Photoshop 2023, different colors were selectively applied to the different arteries and branches to define each of them. This color enhancement was performed to highlight the architecture of the different branches of the MCA. The areas of the camel brain supplied by these branches were determined by integrating previous research on the sulci and gyri in camel brains (20–22).

## 3. Results

The CAC, located at the base of the brain within the middle cranial fossa, acts as a pooling site for the blood coming from the RERM and basilar artery before it is distributed to the brain. The RERM gives rise to the paired RCA and CCA that, at their origin, represent the narrowest point of the CAC (Figure 1).

**Figure 2:**
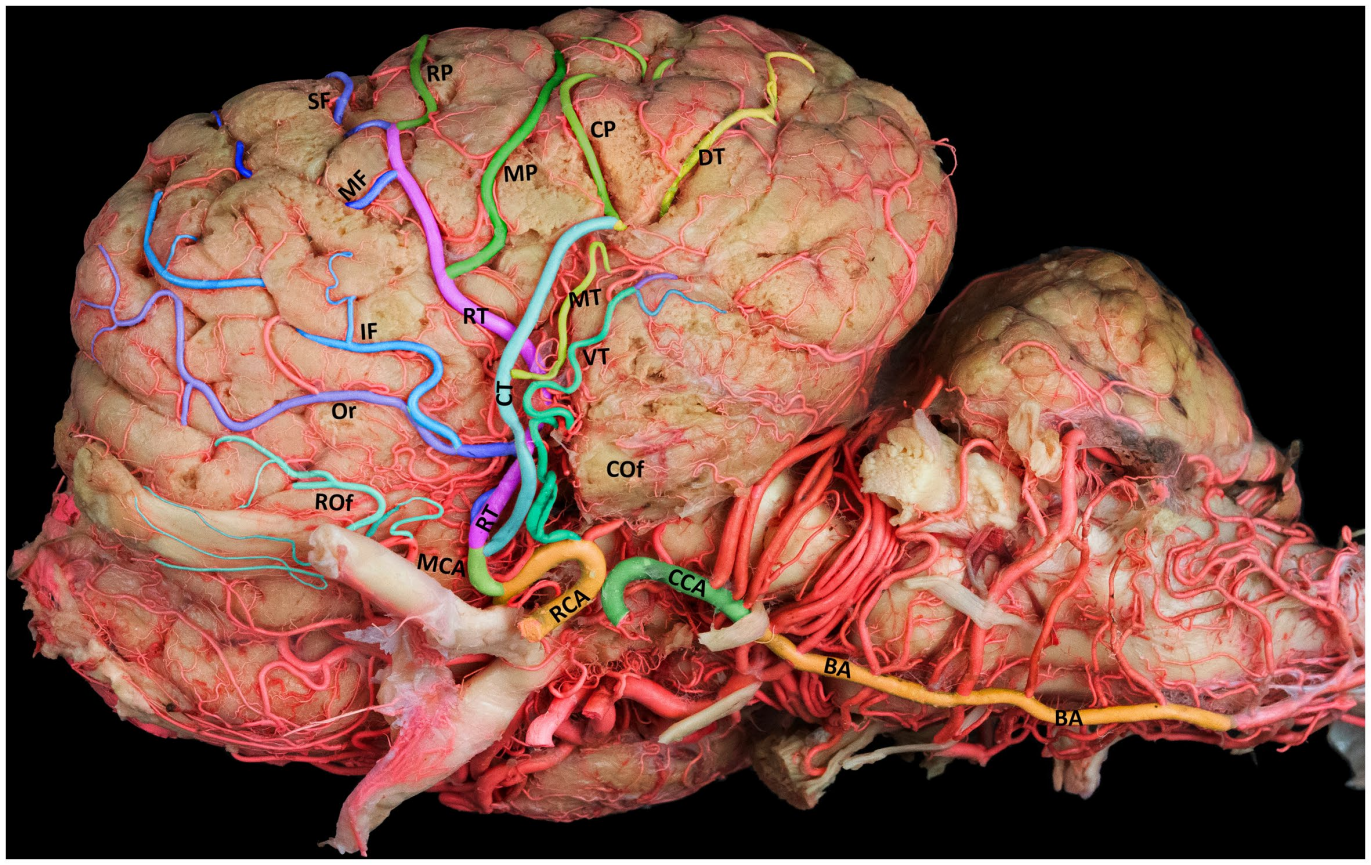
Lateral view of the left cerebral hemisphere, illustrating the standard branching structure of the MCA. BA, basilar artery; CCA, caudal communicating artery; RCA, rostral cerebral artery; MCA, middle cerebral artery; RT, rostral trunk; CT, caudal trunk; Rof, rostral olfactory artery; Cof, caudal olfactory artery; Or, orbital artery; SF, superior frontal artery; MF, middle frontal artery; IF, inferior frontal artery; RP, rostral parietal artery; MP, middle parietal artery; CP, caudal parietal artery; DT, dorsal temporal artery; MT, middle temporal artery; VT, ventral temporal artery.

**Figure 3:**
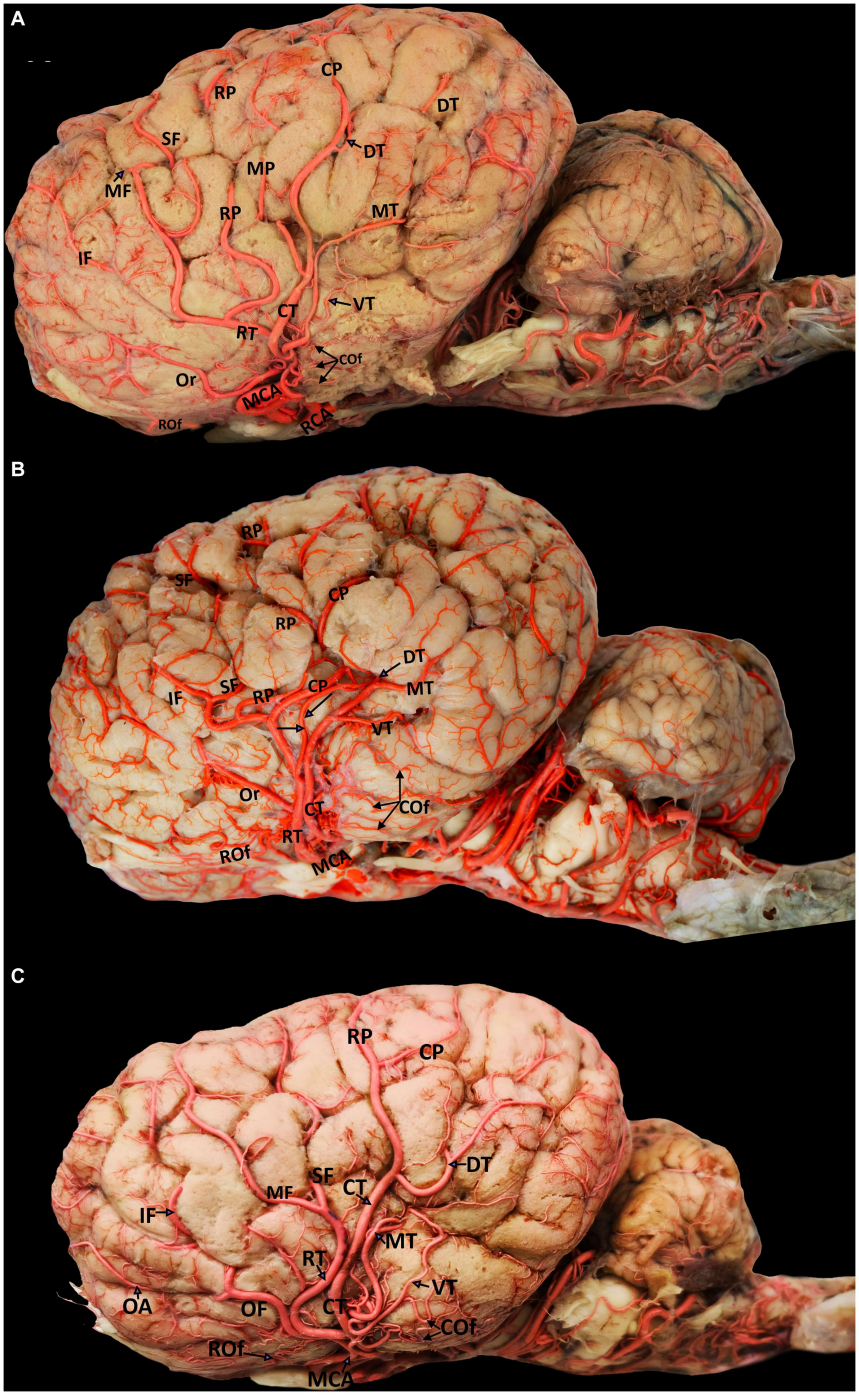
Visual representation of the diverse origins of cortical branches emerging from various segments of the middle cerebral artery (left lateral view). Parts **(A-C)** include the following labels: MCA, middle cerebral artery; RCA, rostral cerebral artery; RT, rostral trunk; CT, caudal trunk; OF, orbitofrontal trunk; Rof, rostral olfactory artery; Cof, caudal olfactory artery; Or, orbital artery; SF, superior frontal artery; MF, middle frontal artery; IF, inferior frontal artery; RP, rostral parietal artery; MP, middle parietal artery; CP, caudal parietal artery; DT, dorsal temporal artery; MT, middle temporal artery; VT, ventral temporal artery.

The MCA originates from the RCA, rostral to the origin point of the choroidal artery. It supplies blood to several deep brain structures, including most of the cerebral lateral surface and the temporal pole of the brain from its base. The MCA travels through the Sylvian fissure before branching into various regions on the lateral surface of the camel brain. Our findings revealed that, in 60% of the examined hemispheres, the MCA originated as a singular branch from the RCA (Figures 2, 3A,C). However, in 40% of the samples, the MCA appeared as two branches of the RCA (Figure 3B).

The MCA has a unique anatomical route in the camel brain, diving into the deep sulci of the cerebral hemispheres to supply blood to the lateral and dorsal aspects of the temporal, parietal, and frontal lobes. Unlike its rostral and caudal counterparts, the MCA follows a superficial path around the curvature of the brain. The MCA gives rise to several deep branches that supply blood to the basal ganglia and the thalamus. It moves dorsally upward through the Sylvian fissure from the base of the brain to the lateral edge of the cerebrum. The main trunk of the MCA traverses the insular cortical tissue within the lateral Sylvian fissure, where it branches into the rostral and caudal trunks (Figure 2) that further extend to numerous cortical branches covering the convex superolateral surface of the cerebral hemisphere. Each of these branches travels toward a certain area of the cerebrum and supplies blood to that region of the brain (Figure 2). We observed numerous tiny branches supplying blood to the piriform lobe and olfactory tract, with blood emerging from the early segment of the main trunk of the MCA.

The cortical branches include the rostral and caudal olfactory arteries and the orbital, superior frontal, middle frontal, inferior frontal, rostral parietal, middle parietal, caudal parietal, dorsal temporal, middle temporal, and ventral temporal arteries. The first branch of the MCA is the rostral olfactory artery. Following its departure from the main stem of the MCA, the rostral olfactory artery travels toward the rostral portion of the lateral rhinal sulcus. Its terminal branches serve the area around the rhinal sulcus and olfactory fissure, including the olfactory tract and the prorean gyrus of the frontal lobe. Our study revealed that the rostral olfactory artery originated independently from the RCA in 80% of the cerebral hemispheres studied (Figures 2, 3A–C) and from the rostral trunk of the MCA in 20% of the hemispheres (Table 1).

**Table 1:** Origin and frequency of branches of the middle cerebral artery in dromedaries.

Branches of MCA	Origin	(%) of origin
Rostral olfactory	RCA	80%
	Rostral trunk of MCA	20%
Caudal olfactory	RCA	35%
	Caudal trunk of the MCA	65%
Orbital artery	Rostral trunk of MCA	35%
	Arises from a common trunk with the inferior frontal artery, known as the orbitofrontal trunk, which originates from the rostral trunk of the MCA	65%
Superior frontal	Rostral trunk of MCA	60%
	Common trunk with middle frontal and rostral parietal arteries	5%
	Common trunk with inferior frontal and rostral parietal arteries	5%
	Common descent with the middle frontal artery from the rostral trunk of the MCA	30%
Middle frontal	Rostral trunk of MCA	65%
	Shared descent with superior frontal artery from MCA’s rostral trunk	30%
	Emerges from a common trunk (with superior frontal and rostral parietal arteries) from MCA’s rostral trunk	5%
Inferior frontal	Common trunk with orbital artery from the rostral trunk of the MCA	65%
	Rostral trunk of MCA	30%
	Common trunk with superior frontal and rostral parietal arteries from MCA’s rostral trunk	5%
Rostral parietal	Rostral trunk of MCA	60%
	Caudal trunk of MCA	30%
	Common trunk with superior and inferior frontal arteries	5%
	Common trunk with middle and superior frontal arteries	5%
Middle parietal	Caudal trunk of MCA	60%
	Rostral trunk of MCA	40%
Caudal parietal	Caudal trunk of MCA	60%
	Rostral trunk of MCA	40%
Dorsal temporal	Caudal trunk of MCA	60%
	Common trunk with ventral and middle temporal arteries, arising from MCA’s main trunk	30%
	Shared descent with the middle temporal artery	10%
Middle temporal	Appears in close proximity to the dorsal temporal branch	10%
	Common trunk with the dorsal and ventral temporal arteries	30%
	Caudal trunk of MCA	60%
Ventral temporal	Caudal trunk of MCA	60%
	Common trunk with the dorsal and middle temporal arteries	30%
	RCA	10%

The first column lists the different branches of the middle cerebral artery (MCA). The second column describes the origin of each branch, detailing any shared trunks with other arteries and their emergence from the MCA or the Rostral Cerebral Artery (RCA). The third column provides the percentage occurrence of each origin, as observed in our study.

As shown in Figures 3A–C, the caudal olfactory artery emerged directly from the RCA in 35% of the cerebral hemispheres and detached independently from the caudal trunk of the MCA in 65% of the samples (Table 1). The terminal branches of the caudal olfactory artery supply blood to the cortex above the caudal portion of the lateral rhinal sulcus as it descends.

**Figure 4:**
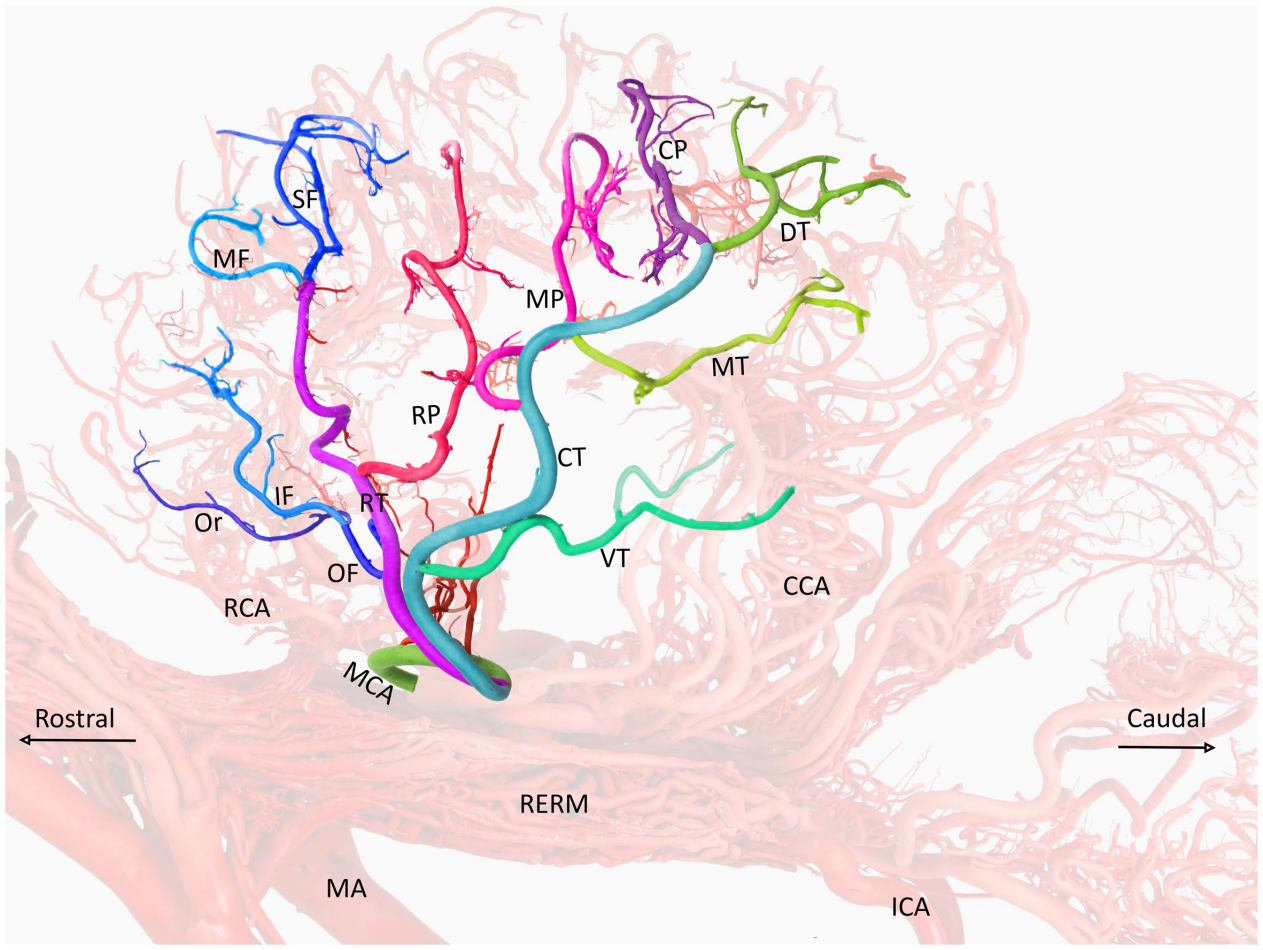
A visual representation of the middle cerebral artery and its color-coded branches (left lateral view) in the dromedary brain. MCA, middle cerebral artery; RCA, rostral cerebral artery; CCA, caudal cerebral artery; MA, maxillary artery; RERM, rostral epidural rete mirabile; ICA, internal carotid artery; OF, orbitofrontal trunk; RT, rostral trunk; CT, caudal trunk; Or, orbital artery; SF, superior frontal artery; MF, middle frontal artery; IF, inferior frontal artery; RP, rostral parietal artery; MP, middle parietal artery; CP, caudal parietal artery; DT, dorsal temporal artery; MT, middle temporal artery; VT, ventral temporal artery.

In 35% of the hemispheres, the orbital artery arose directly from the rostral trunk of the MCA (Figures 3A,B). In 65% of the samples, the inferior frontal and orbital arteries share a common trunk called the orbitofrontal trunk, which arises from the rostral trunk of the MCA (Figures 2, 3C, 4, and Table 1). Several branches of the orbital artery supply blood to the cerebral cortex, located above the pre-Sylvian sulcus and beneath the diagonal sulcus. The final branches supply blood to the cortex above the coronary sulcus.

The inferior frontal artery travels in the direction of the diagonal sulcus, giving off its first branch into the sulcus; the remaining branches then disperse across the surface of the rostral ectosylvian and rostral suprasylvian sulci on their way to the coronary sulcus and fornix. In 65% of samples, the inferior frontal artery shared a common trunk with the orbital artery from the rostral trunk of the MCA (Figures 2, 3C, 4). It originated directly from the rostral trunk of the MCA in 30% of the studied hemispheres (Figure 3A). We observed that the inferior frontal, superior frontal, and rostral parietal arteries arose in 5% of the hemispheres from a shared common trunk that emerged from the rostral trunk of the MCA (Figure 3B and Table 1).

The middle frontal artery was present in 80% of the examined samples. The terminal branches of the middle frontal artery spread across the surface of the cortex of the middle ectosylvian and middle suprasylvian sulci. The middle frontal artery arose directly from the rostral trunk of the MCA in 65% of hemispheres (Figure 3A). Additionally, in 30% of the samples, the middle and superior frontal arteries shared a common descent from the rostral trunk of the MCA (Figures 3C, 4). Interestingly, in a small percentage (5%) of hemispheres, the middle frontal, superior frontal, and rostral parietal arteries emerged from a shared common trunk that originated from the rostral trunk of the MCA (Figure 2 and Table 1).

The superior frontal branch originates from the main trunk of the MCA around the rostral ectosylvian sulcus. It then proceeds to ascend toward the initial segment of the middle suprasylvian sulcus before continuing its upward course toward the cruciate sulcus. The terminal branches of this artery supply the medial surface of the upper portion of the frontal lobes.

We observed that the superior frontal artery had varying origins in the investigated hemispheres. In 60% of hemispheres, the superior frontal artery arose directly from the rostral trunk of the MCA (Figure 3A). In 5% of the samples, the middle frontal and rostral parietal arteries shared a common trunk (Figure 2). Another 5% of the studied hemispheres showed that the superior frontal artery shared a common trunk with the inferior frontal and rostral parietal arteries (Figure 3B). Lastly, in 30% of the samples, the superior frontal artery had a common descent with the middle frontal artery from the rostral trunk of the MCA (Figures 3C, 4, and Table 1).

The blood vessel that climbs through the Sylvian fissure and progresses toward the parietal lobe is divided into three distinct branches. These branches were identified as rostral, middle, and caudal parietal branches. The rostral parietal branch extended from the middle ectosylvian sulcus to the middle suprasylvian sulcus. Its terminal branches supply blood to the cortical area beneath the marginal sulcus, with some of these branches also supplying blood to the rostral ectomarginal sulcus.

We found that the rostral parietal artery directly arose from the rostral trunk of the MCA in 60% of the samples (Figures 3A, 4). In 30% of hemispheres, it originated directly from the caudal trunk of the MCA (Figure 3C). In 5% of the studied hemispheres, the rostral parietal artery shared a common trunk with the superior and inferior frontal arteries (Figure 3B), and in 5% of the samples, the rostral parietal artery had a common trunk with the middle and superior frontal arteries (Figure 2 and Table 1).

The middle parietal artery was observed in 80% of the examined samples (Table 1). This artery supplies blood to the middle suprasylvian sulcus, middle segment of the rostral ectomarginal sulcus, and central region of the marginal sulcus. We found that the middle parietal artery directly originated from the caudal trunk of the MCA in 60% of the samples (Figures 3A, 4), while it was derived from the rostral trunk of the MCA in 40% of the samples (Figure 2).

The caudal parietal branch reaches the middle ectosylvian and caudal suprasylvian sulci, and its terminal branches supply the areas of the marginal and caudal ectomarginal sulci. In our study. In 60% of the samples, the caudal parietal artery directly originated from the caudal trunk of the MCA (Figures 2, 3A,C, 4). In the remaining 40% of the samples, it originated directly from the rostral trunk of the MCA (Figure 3B and Table 1). The temporal branches of the MCA, namely the dorsal, middle, and ventral temporal arteries, supply blood to the caudolateral surface of the cerebral hemispheres, descending at various levels. In 60% of the samples, the dorsal temporal artery originated directly from the caudal trunk of the MCA (Figures 2, 3A–C, 4). In 30% of the samples, the dorsal temporal artery shared a common trunk with the ventral and middle temporal arteries, arising caudally from the main trunk of the MCA. In the remaining 10% of hemispheres, the dorsal temporal artery shared a common descent with the middle temporal artery. The dorsal temporal branch descended into the caudal suprasylvian sulcus and ascended into the upper hemisphere. Some of its terminal branches extended to the marginal and endomarginal sulci. The dorsal temporal branch is commonly regarded as the thickest cortical branch of MCA.

The middle temporal artery traverses the caudal ectosylvian sulcus, while its final branches reach the caudal suprasylvian sulcus and caudal ectomarginal sulcus. Some terminal branches reach and supply blood to the occipital lobes. In our study, the middle temporal artery appeared in close proximity to the dorsal temporal branch in 10% of the samples. In 30% of the samples, it shared a common trunk with the dorsal and ventral temporal arteries. In the remaining 60% of the hemispheres, the middle temporal branch emerged directly from the caudal trunk of the MCA (Figures 2, 3A–C, 4, and Table 1).

The ventral temporal artery supplies blood to the region between the caudal rhinal and ectosylvian sulci, whereas its terminal branches reach the rostral portion of the occipital lobe. In our samples, the ventral temporal artery originated from the caudal trunk of the MCA in 60% of the samples (Figures 3A–C, 4). In another 30% of the hemispheres, the ventral temporal artery shared a common trunk with the dorsal and middle temporal arteries. In 10% of the samples, an independent ventral temporal artery emerged directly from the RCA (Figure 2 and Table 1).

## 4. Discussion

To our knowledge, there is no systematic study on the MCA and its branches in the camel brain. A few studies have only briefly mentioned the origin of the MCA, which emerges from the RCA (3, 4, 18). To gain a deeper understanding of the MCA and its branches in dromedaries, we conducted a comprehensive study. Accurately identifying and defining the branches of the MCA in camels presents a substantial challenge owing to the lack of previous studies. To fill this knowledge gap, we evaluated the branches of the MCA according to the regions they supplied blood to and conducted a comparative analysis between the MCA branches in the dromedary and those observed in the following other animal species: pigs (8), bison (9), goats (12), cattle (13), deer (10), and elks (11).

According to the published results, several factors may influence the pattern of MCA division, including the systematic nomenclature, location, and arrangement of the cerebral sulci on the gyri. In animals, the sulci and gyri are arranged differently on the surface of the cerebral cortex, and this may influence the route and division of the cortical branches of the MCA (19).

The cerebral sulci on the brain’s surface, play a significant role in shaping the branching patterns of the MCA, which must navigate through these sulci to supply blood to the brain tissue. The depth and complexity of these sulci can influence how the MCA branches out to supply blood to the brain tissue. To ensure enough blood flow to all locations, the MCA may need to branch out in more complex ways if the sulcal patterns are deeper or more complex (19, 23).

The study of Shalom et al. (23) explains that the MCA, located in the Sylvian fissure, has to adapt to the changing geometry of the brain’s surface while constructing an efficient network for blood supply. This adaptation process results in a unique, non-redundant arterial system that is sensitive to acute occlusions or blockages. The study also suggests that the dynamics of brain folding condition the development of arterial connections, leading to a network that lacks loops and may have a limited response to acute occlusions. This dynamic nature of sulcal patterns further complicates the relationship between sulcus coverage and MCA branching patterns.

While our understanding of this relationship is still evolving, it is clear that the configuration of sulci on the mammalian cortical surface has a significant impact on the MCA’s division pattern. This understanding is based on our ongoing study on the gyri and sulci of the camel brain (Al Aiyan et al., unpublished). To provide accurate anatomical descriptions of the MCA branches, their positions were connected to the gyri and sulci of the camel brain, which have been previously studied by several researchers (20–22).

Considering these variations, our study presented findings on the camel cortical branches of the MCA, encompassing their course, division, and variation. We compared our findings with the results reported in the literature. This approach enabled us to create a comprehensive map of the MCA branches in the camel brain that could be a resource for future research and clinical applications. Despite the lack of literature on this subject, we were able to provide a consistent and scientifically accurate description of the MCA branches using this approach. To identify these branches based on their area of supply and observe their origin and distribution, it is necessary to track them to the cerebral convexity after dissecting the insular segment of the MCA.

Compared to the rostral and caudal cerebral arteries, the MCA is the brain’s most complicated artery. Our study revealed that the MCA in camels has a greater number of cortical branches and provides blood supply to a larger region of the cerebral hemispheres, consistent with the results of previous studies on the MCA in goats (12), cattle (13), pigs (8), and bison (9).

We observed that the main trunk of the MCA ascended dorsally from the base of the brain through the lateral Sylvian fissure, wound around the piriform lobe, and continued in front of the rostral boundary to the lateral edge of the cerebrum.

According to our observations of the dromedary brain, the MCA emerges directly from the RCA and can have one or more sites of origin. In contrast, some authors assert that the MCA in camels arises from the terminal portion of the internal carotid artery (24, 25). This contradicts our observations that the internal carotid artery contributes to the formation of the RERM. In our study, we observed that only two arteries emerged from the rete and contributed to the formation of the circle of Willis: the CCA and RCA. In cattle and buffaloes, the MCA is the main branch of the RCA (18, 26). However, in goats and sheep, the internal carotid artery divides into the rostral and caudal primary branches. The rostral primary branch is the larger of the two, giving rise to the MCA at the Sylvian fissure and continuing further into the RCA (27–30).

According to Kapoor et al. (30), the rostral and middle cerebral arteries are directly separated from the internal carotid arteries in dogs. In dogs, most of the internal carotid artery’s rostral division continues as the MCA, whereas the smaller RCA winds medially, just dorsolaterally to the optic chiasm (30). Cats have a thick MCA that arises from the midline rostral division of the internal carotid artery and takes a sharp lateral turn toward the Sylvian fissure (31). According to Ozgel and Dursun (32), the internal carotid artery in donkeys splits into the CCA and the RCA inside the cranial cavity, with the MCA branching from the RCA instead of continuing to the internal carotid artery. In contrast to the results reported by Nanda (33) in horses, the internal carotid artery is thought to branch off into the CCA and continue for a short distance, terminating at the rostral and middle cerebral arteries.

Our study revealed that although the MCA typically descended as a single trunk in 60% of the hemispheres, it emerged as two branches from the RCA in 40% of the samples. Skoczylas et al. (34) reported that the MCA in sheep descended as a single artery in 93.3% of the samples, while having two independent origins in 6.7% of the samples.

The MCA typically descends from the RCA in red deer, goats, fallow deer, and European otters (12, 35, 36). Multiple origins of the MCA have been identified in some animals in several studies, including horses (15, 16), roe deer (17), and cattle (18). According to a study by Kielyka–Kurc et al. (14), three Bactrian camels, two dromedaries, and two llamas had a single MCA, but two llamas and one guanaco had two MCAs on each side of the brain. Three Bactrian, one dromedary, and one guanaco camels were observed to have three MCAs. They also observed that two wapitis, five fallow deer, and six reindeer had two points of origin of the MCA, either on the left or right side, or bilaterally. Further, it was observed that the two origins of the MCA were not always of identical thickness. According to Brudnicki et al. (37), deer have a thick MCA, and the majority of blood flow from the RCA is collected by the MCA, resulting in a thinner RCA and branching of the MCA.

Our research showed that the MCA has numerous cortical branches with varying percentages of origin from various segments of the MCA. The olfactory artery, namely the rostral and caudal olfactory arteries, supplies blood to the piriform lobe and olfactory tract. We observed that in 80% of the samples, the rostral olfactory artery was separated from the RCA, whereas in 20% of the samples, it was detached from the rostral trunk of the MCA (Table 1). Regarding the caudal olfactory artery, in 65% of the samples, these branches originated from the caudal trunk of the MCA, whereas in 35%, they originated from the RCA (Table 1). Skoczylas et al. (13) found that in cattle, the rostral olfactory artery originated independently of the RCA in 45% of the studied samples and shared a common trunk with the orbital artery in 5% of the samples. In 25% of the examined samples, the rostral olfactory artery shared a common trunk with the orbital and ventral frontal branches, whereas in the remaining 25%, it shared a common trunk with the orbital, ventral, and dorsal frontal arteries. In contrast, the caudal olfactory artery originates from the main stem of the MCA in 55% of the samples, whereas in 20% of the samples, it shares its origin with the ventral temporal artery. In 15% of the hemispheres studied, the caudal olfactory artery shares a common trunk with the middle and dorsal temporal arteries, whereas in the remaining 10%, the caudal olfactory artery shares a common descent with the ventral, middle, and dorsal temporal branches (13).

The cortical branches of the MCA extend slightly deeper into the cerebral sulcus to supply blood to the frontal, parietal, and temporal lobes on the lateral and dorsal sides of the brain. We found that the frontal lobes were supplied by the superior, inferior, and middle frontal arteries, whereas the parietal lobes were supplied by the rostral, middle, and caudal parietal arteries. Several researchers have overlooked or considered the middle frontal and middle parietal arteries as small branches of the other frontal and parietal arteries in pigs (8), bison (9), goats (12), and cattle (13). The middle frontal and middle parietal arteries in our study were distinct thick branches of various origins that supplied blood to the middle region of the frontal and parietal lobes of the dromedary.

In a study by Skoczylas et al. (13) on the blood supply in the cattle brain, they reported that the ventral, middle, and dorsal temporal branches followed a common descent in 25% of the hemispheres, and the dorsal, middle, and ventral temporal arteries and the rostral and caudal parietal arteries followed a common descent in 30% of the hemispheres. They also observed that the ventral, middle, and dorsal temporal arteries shared a common trunk with the caudal olfactory artery in 10% of the cattle, whereas a shared descent of the caudal olfactory artery and ventral temporal branch occurred in 20% of the samples. A single trunk produced both the middle and dorsal temporal branches in 15% of the samples (13). All the dromedaries in our study had three temporal arteries. We also observed changes in the percentages of the dorsal, middle, and ventral temporal arteries originating from various segments of the middle cerebral arteries (Figures 2, 3A–C, 4).

Our study found that the thalamus and basal ganglia were supplied by small, deeply penetrating branches of the MCA in the dromedaries reflecting the high metabolic demand of these regions. Similar findings have also been reported for MCA distribution in pigs (8), goats (12), and cattle (13). The MCA in cattle distributes branches to the tuberculum olfactorium, piriform lobe, and lateral and dorsal aspects of the cerebral hemisphere; the deeply penetrating branch gives rise to branches extending to the medial aspect of the basal ganglia, with its terminal branches anastomosed with the rostral and caudal cerebral branches (38). Interruptions in the blood flow to these regions, such as those caused by stroke or vascular abnormalities, can lead to significant neurological deficits, including sensory loss and motor dysfunction (39). While such correlations have not been clearly studied in dromedaries, it is likely that similar relationships may exist given the fundamental roles of the thalamus and basal ganglia in neural functioning.

In dogs, the MCA has numerous secondary branches, including substantial proximal branches that supply blood to the olfactory structures, and distal branches that follow the Sylvian fissure, encircle the neopallium, cross the gyri, and terminate in the sulci of the cerebral hemisphere (30). The MCA in cats provides blood supply to the olfactory bulb, olfactory tract, tuberculum olfactorium, and piriform region and provides cortical branches that radiate rostrally and laterally into the neocortex (31). The authors of the two previous studies reported that the RCA and CCA were anastomosed with the terminal branches of the MCA in dogs and cats. Our research reveals distinct differences between dromedaries and other animal species in the branching patterns of the MCA. We think that these variances could be a result of the camels’ unique adaptations to the harsh desert environment. For instance, camels’ brains have a special cooling system that keeps it from overheating even in extremely hot conditions. This cooling system relies on a complex network of blood vessels, including the MCA, which may explain the observed variations in its branching patterns.

## 5. Conclusion

This groundbreaking study fills a significant gap in literature since, to our knowledge, it presents the first thorough investigation of the MCA and its branches in camels. We successfully identified several arteries that cross the frontal and parietal lobes, including the middle frontal and middle parietal arteries. These arteries have not been reported in previous studies of other animal species. The identification of these newfound arteries advances our knowledge about the brain and blood flow in dromedaries. For example, the middle frontal and middle parietal arteries, which were discovered for the first time in our study, may have a significant impact on the function of particular brain regions in the camel and may increase their sensitivity to certain neurological disorders. Our study’s larger sample size allowed for the consideration of different variations in the branches of the MCA. These variations, including the origins of each branch and their shared trunks with other arteries, as well as the percentage occurrence of each origin, are comprehensively detailed in Table 1. Additionally, our results describe the early branches of the MCA, including the olfactory arteries, as well as numerous cortical branches that divide into the frontal, parietal, and temporal branches. This study lays the foundation for future studies on the arterial supply to the brains of dromedaries by highlighting their distinct anatomical features.

## Data availability statement

The raw data supporting the conclusions of this article will be made available by the authors, without undue reservation.

## Ethics statement

The animal study was reviewed and approved by Animal Research Ethics Committee of the United Arab Emirates University.

## Author contributions

AA: study design, conception, anatomical dissection, analysis, acquisition of data, photography and photo editing, drafting of the manuscript, and revision of the manuscript. RB: analysis of data and drafting of the manuscript. All authors contributed to the article and approved the submitted version.

## Funding

The study was funded by a project grant from the United Arab Emirates University, UAE (Grant Code 31F134).

## Conflict of interest

The authors declare that the research was conducted in the absence of any commercial or financial relationships that could be construed as a potential conflict of interest.

## Publisher’s note

All claims expressed in this article are solely those of the authors and do not necessarily represent those of their affiliated organizations, or those of the publisher, the editors and the reviewers. Any product that may be evaluated in this article, or claim that may be made by its manufacturer, is not guaranteed or endorsed by the publisher.

## Footnotes

^1^Al Aiyan A. Exploring the Exterior Architecture of the Dromedary Camel’s Brain. (2019) [unpublished].
